# Anthocyanin Encapsulated Nanoparticles as a Pulmonary Delivery System

**DOI:** 10.1155/2022/1422929

**Published:** 2022-09-10

**Authors:** Madumani Amararathna, David W. Hoskin, H. P. Vasantha Rupasinghe

**Affiliations:** ^1^Department of Plant, Food, and Environmental Sciences, Faculty of Agriculture, Dalhousie University, Truro, NS, Canada B2N 5E3; ^2^Department of Pathology, Faculty of Medicine, Dalhousie University, Halifax, NS, Canada B3H 4H7

## Abstract

Anthocyanins are known for their therapeutic efficacy for many human diseases, including cancer. After ingestion, anthocyanins degrade due to oxidation and enzymatic breakdown, resulting in reduced therapeutic efficacy. Direct delivery to target tissues and entrapment of anthocyanins increases their stability, bioavailability, and therapeutic efficacy. The objective of the present study was to develop a direct delivery system of anthocyanins into pulmonary tissues via encapsulated nanocarriers. A cyanidin-3-*O*-glucoside (C3G)-rich anthocyanin extract was prepared from well-ripened haskap (*Lonicera caerulea* L.) berries (HB) and encapsulated in three different polymeric nanocarrier systems: polyethylene glycol-poly(lactide-*co*-glycolide), maltodextrin, and carboxymethyl chitosan (CMC). The anthocyanin encapsulation efficiency was significantly higher in CMC (10%) than in the other two polymers. The cytotoxicity and cytoprotective effect of HB anthocyanin-encapsulated CMC (HB-CMC, 4 *μ*g of C3G equivalent anthocyanin in 2 mg/mL nanoparticle) and anthocyanin-free CMC (E-CMC, 2 mg/mL) were tested for cytotoxicity using human normal lung epithelial BEAS-2B cells. The CMC nanoparticles were not cytotoxic for BEAS-2B cells. The HB-CMC nanoparticles reduced carcinogen-induced oxidative stress in BEAS-2B cells and restored the expression of superoxide dismutase and glutathione peroxidase enzymes. The HB-CMC nanoparticles also reduced carcinogen-induced DNA single-strand breaks and alkaline-labile sites but not the double-strand breaks. The E-CMC, HB-CMC (28 *μ*g C3G equivalent/mouse/day for six days), or the same dose of free HB anthocyanin was administered to A/JCr mice through a nose-only passive inhalation device. C3G and its metabolites, cyanidin, peonidin-3-*O*-glucoside, and cyanidin-3-*O*-glucuronide, were detected by UPLC/ESI/Q-TOF-MS in the lungs of mice after one hour of exposure. Therefore, the CMC could be a promising noncytotoxic candidate to encapsulate HB anthocyanin. Direct delivery of anthocyanin to lung tissues enhances tissue retention, slows phase 2 metabolism, and improves therapeutic efficacy.

## 1. Introduction

Natural and synthetic biopolymers are being widely used in synthesizing nanoparticles for pharmaceuticals. Polyethylene glycol-poly(lactide-*co*-glycolide) (PEG-PLGA), maltodextrin (MDX), chitin and its derivatives, gelatin, pectin, and cellulose are some of the popular biopolymers [1, 2]. Besides enhancing the bioavailability, nanocarriers help the encapsulants escape from enzymatic digestion and phagocytosis. Anthocyanin, composed of two phenyl benzo pyrylium (flavylium) salt derivatives and hydroxyl and methoxy groups, possesses anticancer, antioxidative, and anti-inflammatory properties [3–5]. Once ingested, anthocyanins undergo various structural modifications due to acidic pH in the stomach, enzymatic digestion, and microbial biotransformation, resulting in poor therapeutic efficacy as a dietary source [6–8]. For instance, only 1.7% of cyanidin 3-*O*-glucoside (C3G) was bioavailable after administering 500 mg/kg C3G to C57BL/6 J mice. Minor levels of C3G were detected in mouse tissues, 28.3 nmol h/g in the lung, 3,655 nmol h/mL in urine, and 35.3 nmol h/g in the liver within one hour of ingestion [9]. In healthy adults, serum C3G levels were 0.31 *μ*mol h/L after 48 h of consuming 500 mg C3G. A higher bioavailability (about 12%) of C3G and its metabolic derivatives was reported in humans [10]. Direct delivery of anthocyanins into target tissues avoiding the oral route enables therapeutic dose provision and enhances therapeutic efficacy in clinical settings.

Haskap berry (HB) is rich in anthocyanins, especially C3G. Anthocyanin-rich HB reduced carcinogen-induced DNA damage, oxidative stress, and lung tumorigenesis [11–13]. PEG-PLGA, MDX, and carboxymethyl chitosan (CMC) were selected to encapsulate C3G-rich anthocyanins extracted from HB. The amphiphilic PEG-PLGA di-block polymer resists opsonization and phagocytosis, thereby sustaining drug circulation in the blood [14–16]. MDX is a carbohydrate-based polymer with D-glucose units linked by *α*(1→4) or *α*(1→6) glycosidic bonds. MDX is inexpensive and widely used in the food industry due to its good biocompatibility and biodegradability [17]. Chitosan-based encapsulants have various applications in the delivery of drugs for the treatment of cancer, gastrointestinal diseases, and pulmonary diseases. Deacetylation of chitosan generates water-soluble CMC, which has carboxyl groups [18]. The CMC nanoparticles generally have a positive surface charge and mucoadhesive properties, facilitating drug release by adhering to the mucous membrane [19]. Therefore, we hypothesized that direct delivery of HB anthocyanins to lung tissues will enhance its bioavailability and therapeutic efficacy against lung carcinogenesis. The present study demonstrates encapsulation of the anthocyanins extracted from HB in PEG-PLGA, MDX, and CMC by emulsification-solvent evaporation, antisolvent nanoprecipitation, and ionic gelation with CaCl_2,_ respectively. The primary objective of the study was to facilitate the direct delivery of HB anthocyanins into pulmonary tissues via encapsulated nanocarriers.

## 2. Materials and Methods

### 2.1. Materials

The nontumorigenic bronchial epithelial cell line, BEAS-2B (ATCC® CRL-9609™), was purchased from the American Type Culture Collection (ATCC; Manassas, VA, USA). LHC-9 bronchial epithelial growth medium (cat. no. 12680013), Dulbecco's Modified Eagle's Medium (DMEM, cat. no. A1443001), and P2 grade filter papers (cat. no. 09-805-5C) were purchased from Thermo Fisher Scientific, Ottawa, ON, Canada. Dulbecco's phosphate-buffered saline (PBS, cat. no. D8537), fibronectin from human plasma (cat. no. F2006), bovine serum albumin (BSA, cat. no. A8022), dimethyl sulfoxide (DMSO, cat. no. 2438), 0.25% Trypsin – 0.53 mM ethylenediaminetetraacetic acid (cat. no. T3924), Triton X-100 (cat. no. T8787), 2,7-dichlorofluorescein diacetate (DCFDA, cat. no. D6883), polyvinylpyrrolidone (cat. no. P0930), penicillin-streptomycin (cat. no. P0781), phosphatase substrate (cat. no. S0942), phenazine methosulfate (PMS, cat. no. P9625), Folin-Ciocalteu reagent (Folin-C, cat. no. F9252), 2,2 diphenyl-1-1picryl-hydrazyl (DPPH, cat. no. D9132), and 2,3,4-Tris(2-pyridyl)-s-triazine (TPTZ, cat. no. T1253) were purchased from Sigma® Life Science, St. Louis, MO, USA. 4-(Acetoxymethyl)nitrosamino-1-(3-pyridyl)-1-butanone (NNKOAc, cat. no. 167550) was purchased from Toronto Research Chemicals, North York, ON, Canada. Bovine collagen type I (PureCol® 5409) was purchased from Advanced BioMetrix, Carlsbad, CA, USA. 7-Aminoactinomycin D (7AAD, cat. no. 00-6993-50) was purchased from eBioscience Inc. San Diego, CA, USA. PLGA–PEG (5,000 : 7,000, cat. no. 765139), sodium deoxycholate (cat. no. D6750), MDX (dextrose equivalent, 4.0 – 7.0, cat no. 419672), sodium dodecyl sulfate (cat no. L3771), Tween® 80 (cat no. P1754), and dichloromethane (DCM, cat no. 270997) were purchased from Sigma-Aldrich, Oakville, ON, Canada. CMC (cat. no. sc-358091) with a 90% deacetylation degree was purchased from Santa Cruz Biotechnology, Mississauga, ON, Canada. All the other chemicals used were of analytical grades. The anti-phospho-H2AX, anti-H2AX, anti-phospho-Nrf2, anti-Nrf2, superoxide dismutase (SOD), catalase, glutathione peroxidase (GPx), goat anti-mouse IgG, goat anti-rabbit IgG, rabbit anti-goat IgG, and anti-actin antibodies were from Cell Signaling Technology, Danvers, MA, USA. Blue loading buffer (cat. no. B7703S) was purchased from New England BioLabs Inc., Ipswich, MA, USA. The chemiluminescence (ECL) based Clarity™ and Clarity Max™ Western ECL Substrates Kit (cat. no. 1705060) were from Bio-Rad Laboratories Inc., Hercules, CA, USA. The ultrasonic mesh nebulizers (MedPro SONAIR_TM_, Model 705-460) were from A.M.G. Medical Inc., Dalton, Montreal, Canada.

The experiment was conducted in three stages. First, HB anthocyanin-encapsulated nanoparticles were prepared and characterized for their physical and chemical properties. Secondly, their cytoprotective properties were tested in cultured normal lung epithelial cells *in vitro*. Finally, the lung bioavailability of HB anthocyanin-encapsulated nanoparticles was tested in mice following pulmonary delivery through passive inhalation.

### 2.2. Preparation of Anthocyanin-Rich HB Extract

Well-ripened fresh HB (Brix value 14-16%) were obtained from the LaHave Natural Farms (Blockhouse, NS, Canada) and frozen at -20°C. The frozen berries (100 g) were ground in 500 mL deionized water (1 : 5, w/v) in a commercial blender (HBB909, Hamilton Beach Brands Inc., Glen Allen, VA, USA) for 20 min, and the juice was filtered under a vacuum. Then, the juice was purified by solid-phase column chromatography to obtain a sugar-free anthocyanin-rich HB extract (Brix value ≤0.1). The column consisted of brominated styrenic adsorbent with >250 *μ*m particle size, 210 Å porosity, 630 m^2^/g surface area, 43–53% water content, and 780 g/L bulk density. The Brix value was measured by a refractometer. Once the water-soluble sugar fraction was eluted completely, the column-bound nonsugar anthocyanin fraction was eluted with 20%, 70%, and 95% ethanol gradient. The combined anthocyanin-rich mixture was rotary evaporated (Heidolph RotaChill, UVS400-115, Thermo Electron Corporation, Milford, MA, USA), followed by further drying under a gentle nitrogen flow (N-EVAP™ 111, Organomation Associates Inc., Berlin, MA, USA). The concentrated samples were lyophilized (Dura-Dry™ MP FD-14-85BMP1, DJS Enterprises, Markham, Ontario, Canada) under a 3,600 mT vacuum at -20°C for 24 h, and the dried powder was stored at -80°C in amber bottles until use.

### 2.3. Preparation and Characterization of HB Anthocyanin-Encapsulated Nanoparticles

Three types of FDA-approved polymers, PEG-PLGA, MDX, and CMC, were tested at two different polymer to anthocyanin (w/w) ratios to choose the most suitable material for HB anthocyanin encapsulation.

#### 2.3.1. PEG-PLGA Nanoparticles

PEG-PLGA nanoparticles were prepared by the emulsification-solvent evaporation technique [14]. Briefly, HB anthocyanin and PEG-PLGA were dissolved (1 : 10 and 1 : 20 w/w) in 3 mL of DCM at room temperature under continuous stirring for approximately 30 min. This mixture was added to a 12 mM sodium deoxycholate solution (20 mL) on an ice bath, and the mixture was probe-sonicated (model 505 Sonic, Thermo Fisher Scientific Inc., Waltham, MA, USA) at 40% power for 5 min. Then, the organic phase was evaporated entirely, and nanoparticles were separated by centrifugation (Sorvail ST 16, Thermo Fisher Scientific Inc., Waltham, MA, USA) at 14,000×*g* for 10 min at 4°C. The particle pellet was washed twice with deionized water and lyophilized. Anthocyanin-free PEG-PLGA nanoparticles were also prepared following the same method without HB.

#### 2.3.2. MDX Nanoparticles

The MDX nanoparticles were prepared as described by Qui and colleagues [17]. A solution of MDX (5% w/v) was prepared with 0.5% (v/v) Tween 80%. The HB anthocyanin was mixed with MDX solution (1 : 10 and 1 : 20 w/v). While stirring continuously (600 rpm), absolute ethanol (100 mL) was added dropwise to 10 mL of the above suspension. The suspension was centrifuged at 14,000×*g* at 4°C, rinsed with absolute ethanol twice, and lyophilized. The anthocyanin-free MDX nanoparticles were made following the same procedure without HB anthocyanin.

#### 2.3.3. CMC Nanoparticles

**Figure 1:**
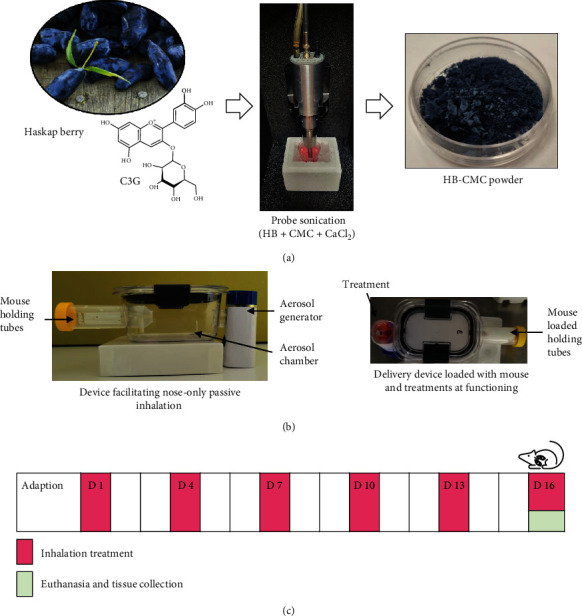
The experimental plan and the timeline of delivering anthocyanins via the respiratory route. (a) Preparation of HB-CMC nanoparticles by ionic gelation. (b) The methodology of delivering nanoparticles via nose-only exposure. The nose-only delivery devices consisted of an ultrasonic mesh nebulizer connected to an aerosol holding chamber connected to a perforated mouse holding chamber. (c) Timeline of pulmonary delivery of anthocyanins. Numbers (D1–D16) represent days from the beginning of the experiment. CMC: carboxymethyl chitosan; HB: haskap berry anthocyanin.

The CMC nanoparticles were prepared based on previously reported methods with a few modifications [20, 21]. A 1 mg/mL solution of CMC solution was prepared, and pH was adjusted (pH 7) and diluted into 0.5 mg/mL. The HB was mixed (1 : 10 and 1 : 20 w/w), and the nanoparticles were synthesized by dropwise addition of CaCl_2_ (1 mg/mL) into 5 mL of the CMC-HB solution while stirring. Then, the mixture was probe-sonicated for 3 min on an ice bath at 40% power, centrifuged at 14,000×*g* for 15 min at 4°C, and the pellet was washed twice. The anthocyanin-free CMC nanoparticles were prepared following the same procedure without HB. All the samples were lyophilized (Figure 1(a)).

### 2.4. Determination of the Chemical Characteristics of Nanoparticles

#### 2.4.1. Anthocyanin Content

The total monomeric anthocyanin content was determined by the pH differential method, which measures the structural transformation of anthocyanins at different pH levels [22]. Briefly, 1 mg PEG-PLGA, MDX, and CMC nanoparticles were dissolved in 2 mL of DCM, deionized water, and 1% formic acid, respectively, sonicated for 10 min at room temperature, and then centrifuged (5,000×*g* for 10 min) to remove structural debris. Each nanoparticle mixture was diluted in pH 1.0 buffer (potassium chloride, 0.025 M) and pH 4.5 buffer (sodium acetate, 0.4 M) in 1 : 10 (v/v) ratio, separately. The absorbance was measured at 520 nm and 700 nm using a plate reader (Tecan Infinite® M200 PRO, Morrisville, NC, USA). All the measurements were carried out in a quadruplicate. According to the Equation (1), the total anthocyanin was calculated.

(1) $$\begin{array}{c} \text{Total anthocyanin }\left(\text{mg C}3\text{G}\frac{\text{equivalant}}{L}\right)=\frac{\left(A\times \text{MW}\times \text{DF}\times 1000\right)}{\left(\varepsilon \times L\right)}, \end{array}$$

where *A* = (absorbance at 520 nm–absorbance at 700 nm at pH 1)–(absorbance at 520 nm–absorbance at 700 nm at pH 4.5); MW is the molecular weight of C3G, 449.2 g/mol; DF is the dilution factor; molar extinction coefficient (*ε*) is 26,900 mol/L/cm; *L* is the path length (1 cm); and 1,000 is the conversion factor from g to mg.

#### 2.4.2. Anthocyanin Encapsulation Efficiency

The anthocyanin encapsulation efficiency was calculated using the following equation:

(2) $$\begin{array}{c} \text{Encapsulation efficiency }\left(\%\right)=\frac{\text{Amount of entrapped anthocyanin in nanoparticles}}{\text{Amount of anthocyanin used to prepare nanoparticles}}*100. \end{array}$$

#### 2.4.3. The Anthocyanin Loading Capacity

The anthocyanin loading capacity was calculated according to the following equation:

(3) $$\begin{array}{c} \text{Loading capacity }\left(\%\right)=\frac{\text{Monomeric anthocyanin in }10\text{ mg of nanoparticles}}{10\text{ mg of nanoparticles}}*100. \end{array}$$

#### 2.4.4. Total Phenolic Content (TPC)

The TPC was tested by Folin-Ciocalteu assay [23]. The anthocyanin-encapsulated nanoparticles were disintegrated, and 20 *μ*L was mixed with 0.2 N Folin-Ciocalteu (100 *μ*L). After a 5 min incubation, 80 *μ*L of 7.5% sodium carbonate solution was added. The plate was incubated for another 2 h at room temperature before reading at 760 nm. TPC was expressed as mg gallic acid equivalent/g dry weight (mg GAE/g DW).

#### 2.4.5. Antioxidant Capacity

The antioxidant capacity was determined by the ferric reducing antioxidant power (FRAP) and the DPPH-free radical scavenging assays.

#### 2.4.6. FRAP Assay

The antioxidant capacity was determined by measuring the electron donation potential of samples, as described by Benzie and Strain and modified by Rupasinghe [24, 25]. Nanoparticles were disintegrated. A working solution consisting of 300 mM acetate buffer (pH 3.6), 20 mM ferric chloride, and 1 mM TPTZ solution (10 : 1 : 1, v/v/v) was made freshly and added (180 *μ*L) to 20 *μ*L of sample or standard in 96-well microplate. The absorbance was measured at 593 nm after a 6-min incubation at room temperature in the dark. The antioxidant capacity was calculated as *μ*M Trolox equivalent/g dry weight (*μ*M TE/g DW).

#### 2.4.7. DPPH Assay

The antioxidant activity of anthocyanin-loaded capsules was determined through the DPPH· radical scavenging assay. The assay was adapted from Blois to perform a 96-well microplate [26]. Briefly, 0.2 mM DPPH· reagent was prepared. Nanoparticles were disintegrated and dissolved in water to a concentration gradient (50, 100, 200, 400, 800, and 1,600 *μ*g/mL), and 150 *μ*L of DPPH· reagent was pipetted into each well containing 150 *μ*L of the sample. The following equation calculated the inhibition percentage:

(4) $$\begin{array}{c} A\text{ntioxidant activity }\left(\%\text{inhibition}\right)=\frac{\left(\text{Ab blank}-\text{Ab sample}\right)}{\text{Ab blank}}\text{X }100, \end{array}$$

where Ab sample is the absorbance value of anthocyanin encapsulated nanoparticles and Ab blank is the absorbance value of HB anthocyanin-free nanoparticles. The antioxidant capacity of HB extracts was expressed as IC_50_, defined as the concentration of tested material required to cause a 50% decrease in initial DPPH· concentration. The IC_50_ value was calculated using the % inhibition vs. antioxidant concentration curve by Microsoft Office Excel Version 14.0.

#### 2.4.8. Zeta Potential

The zeta potential was measured by dynamic laser light scattering using a Zetasizer (Malvern Nano-series, Malvern Instruments Ltd., Malvern, UK). Briefly, the nanoparticles were resuspended in deionized water (1 mg/mL). The surface charge was measured at 25 ± 2°C, using a nominal 5 mW He–Ne laser operating at 633 nm wavelength.

### 2.5. Determination of the Physical Characteristics of Nanoparticles

#### 2.5.1. Transmission Electron Microscopy (TEM)

The shape and surface morphology were examined using TEM images (JEOL 1230 TEM, JEOL USA Inc., Peabody, MA, USA). Lyophilized nanoparticles were dissolved in deionized water and placed on a TEM grid surface. Negative staining was performed with 10% uranyl acetate, and excess fluid was blotted off. The stained samples were air-dried at room temperature, and the TEM grid was then loaded into the transmission electron microscope, and images were taken.

#### 2.5.2. Particle Size

The particle size was measured by dynamic laser light scattering using a Zetasizer in a 1 mg/mL nanoparticle suspension.

### 2.6. Determination of the Cytoprotective Properties of Nanoparticles In Vitro

BEAS-2B cells were cultured in T-75 flasks coated with a mixture of 0.03 mg/mL bovine collagen type I, 0.01 mg/mL BSA, and 0.01 mg/mL fibronectin and dissolved in sterile 1 × PBS. LHC-9 was used as the cell culture medium. Throughout the experiment, the cells were maintained at 37°C, 5% CO_2_, and in a 100% humidified environment (VWR3074, Hampton, NH, USA). All the experiments were conducted between passages 5 and 30. BEAS-2B cells were treated with anthocyanin-free CMC nanoparticles (E-CMC, 2 mg/mL), HB anthocyanin-encapsulated CMC nanoparticles (HB-CMC, 2 mg/mL to provide 4 *μ*g/mL C3G equivalent anthocyanin), and 4 *μ*g/mL of HB for 3 h, separately. The treated cells were washed with 1 × PBS and exposed to 100 *μ*M NNKOAc for another 3 h. The cells with and without NNKOAc exposure were tested to determine the cytoprotective effect of HB-encapsulated nanoparticles in BEAS-2B normal bronchial epithelial cells.

#### 2.6.1. MTS Assay

BEAS-2B cells were cultured at a density of 5 × 10^3^ cells/well in 96-well flat-bottom microplates treated with HB-CBC and E-CMC nanoparticles for 24 h. In the end, 10 *μ*L MTS reagent (1: 20 PMS: MTS) was added to each well and incubated at 37°C for 3 h. The absorbance was measured at 490 nm. The cell viability was calculated as expressed in

(5) $$\begin{array}{c} \text{Cell viability }\left(\%\right)=\frac{\text{Absorbance value of the treated cells}-\text{Blank}}{\text{Absorbance value of the negative control cells}-\text{Blank}}\text{X }100, \end{array}$$

where blank is the absorbance of the tested compounds without cells.

#### 2.6.2. 7-AAD Assay

BEAS-2B cells were plated at 2 × 10^5^ cells/well density in 6-well plates and incubated overnight at 37°C to encourage cell adhesion. Adhered cells were treated with 4 *μ*g/mL anthocyanin-equivalent HB-CMC (2 mg/mL HB-CMC nanoparticles), anthocyanin-free nanoparticles (2 mg/mL), and HB extracts 4 *μ*g/mL for 3 h at 37°C. Then, the treated monolayers were washed carefully with PBS and exposed to 100 *μ*M NNKOAc for another 3 h. Cells and culture supernatants were harvested, combined, and centrifuged at 250×*g* for 5 min. Cells were resuspended in PBS, stained with 2.5 *μ*L of 7-AAD viability staining solution at room temperature, and measured by flow cytometer (Attune NxT Acoustic Focusing Cytometer, Thermo Fisher Scientific Inc., Waltham, MA, USA) at 488 nm excitation laser and 695/40 nm emission filter.

#### 2.6.3. DCFDA Assay

The generation of reactive oxygen species (ROS) was determined using DCFDA. Briefly, cells were cultured at 2 × 10^5^ density/well in 6-well plates and incubated for 24 h. The attached cells were stained with 10 *μ*M DCFDA and dissolved in serum- and phenol red-free DMEM medium for 45 min at 37°C. Stained cells were thoroughly rinsed with 1 × PBS and treated as described above. Then, the treated monolayers were rewashed and exposed to 100 *μ*M NNKOAc for another 3 h. Cells and culture supernatants were harvested, centrifuged at 500×*g* for 5 min, and fixed in 300 *μ*L 1% paraformaldehyde for 15 min at room temperature after washing in 1 × PBS. Fixed cells were washed in PBS and analyzed by flow cytometer at 488 nm excitation laser and 530/30 nm emission filter.

### 2.7. Determination of the Genoprotective Properties of Nanoparticles In Vitro

#### 2.7.1. Comet Assay

DNA damage in cells was evaluated using the alkaline and neutral comet assays [27]. Cells were grown in 6-well plates at a density of 1 × 10^5^ cells/well and treated as explained above. After each treatment, cells were harvested and centrifuged at 125×*g* for 5 min. The cell pellet was washed in cold Ca^2+^ and Mg^2+^ ion-free PBS and resuspended in cold PBS. Agarose (0.7%, w/v) was melted in boiling water and cooled in a 37°C water bath for 20 min. Cells were mixed with molten agarose at room temperature at a ratio of 1: 10 (v/v) and immediately pipetted (30 *μ*L) onto glass slides, and the mixture was gently spread to make a thin layer. The slides were immersed in prechilled lysis solution [2.5 M NaCl, 100 mM EDTA (pH 10), 10 mM Tris base, 1% sodium lauryl sarcosinate, and 1% Triton X-100] and incubated at 4°C for 45 min. For the alkaline comet assay, DNA unwinding was facilitated by dipping the slides in freshly prepared 0.3 M NaOH containing 1 *μ*M EDTA (pH =9) for 20 min in the dark at room temperature, followed by gel electrophoresis at 16 V for 12 min. For the neutral comet assay, slides were immersed in cold neutral electrophoresis solution (100 mM Tris base and 300 mM sodium acetate in 1 L deionized water, pH =9) for 30 min at 4°C in the dark. The gel electrophoresis was carried out in the same neutral electrophoresis solution at 16 V for 25 min. Then, slides were immersed in 70% ethanol for 5 min and air-dried in the dark, followed by staining in CYGREEN® dye (diluted in 1: 1,000 in DI water, v/v) for 30 min at room temperature in the dark. The slides were briefly rinsed three times in DI water and dried before imaging.

#### 2.7.2. *γ*H2A.X Assay

BEAS-2B cells were cultured in 6-well plates (2 × 10^5^ cells/well) and treated with nanoparticles, HB extracts, and NNKOAc, as explained above. Treated cells were harvested, centrifuged at 125×*g* for 5 min, washed once in cold 1 × PBS, and resuspended in cold 0.5 mL PBS to get a single cell suspension. The cells were fixed in 70% ice-cold ethanol and kept at -20°C overnight. The fixed cells were permeabilized in 0.5 mL of 0.25% Triton X-100 dissolved in PBS for 5 min at room temperature. The primary antibody was diluted (1 : 250) in 1% BSA and incubated for 60 min under gentle agitation at room temperature. The cells were rinsed once in PBS and stained with fluorescein-conjugated secondary antibody (1 : 500) for another 45 min in the dark. After rinsing off excess antibodies, cells were resuspended in 0.3 mL PBS and analyzed by flow cytometry at 488 nm excitation laser and 530/30 nm emission filter.

#### 2.7.3. Western Blotting

BEAS-2B cells were collected after the treatments, as previously described, and lysed using ice-cold radioimmunoprecipitation assay lysis buffer containing freshly added protease inhibitors for 15 min on an ice bath. The cell lysates were collected at 14,000×*g* for 15 min at 4°C, and the protein concentration was measured by Bradford assay. Proteins were denatured and equalized using the sample buffer. Equal amounts of protein (20 *μ*g) were loaded into 10% polyacrylamide gels and electrophoresis for 35 min at 200 V and 400 mA. Proteins were transferred to nitrocellulose membranes, and blots were incubated in 5% skim milk prepared in Tween-TBS [0.25 M Tris (pH 7.5), 150 mM NaCl, and 0.2% Tween-20] for 1 h at room temperature to block nonspecific binding. Blots were probed with primary antibodies (1 : 1,000) overnight at 4°C and then washed thoroughly with Tween-TBS (15 min ×3) and probed with HRP-conjugated donkey anti-rabbit or anti-mouse IgG antibody (1 : 5,000) for 1 h. The proteins of interest were visualized by enhanced chemiluminescence in the dark. Uniform protein loading was confirmed by probing with HRP-conjugated rabbit anti-actin antibody.

### 2.8. Lung Bioavailability of Anthocyanins from Nanoparticles

After pulmonary delivery, the bioavailability of anthocyanin encapsulated nanoparticles in mice lungs was evaluated. This study was performed at Dalhousie University's animal care facility, following the University Committee on Laboratory Animals (protocol 2018-007). Female A/JCr albino mice at six weeks were purchased from Charles River Laboratories, Inc., Montreal, QC, Canada.

#### 2.8.1. A/JCr Mice Model

There were three treatment groups: control (E-CMC), HB-CMC, and free HB anthocyanin inhalation. Each group consisted of 5 mice. A nose-only drug delivery device was designed to facilitate passive inhalation of each treatment (Figure 1(b)). An ultrasonic mesh nebulizer was connected to a bisphenol A-free polypropylene chamber (300 mL volume) at one end, and the opposite end of the chamber was connected to a perforated (50 mL) polypropylene tube to hold the mouse. Nanoparticles and the HB anthocyanins were dissolved separately in sterilized deionized water (4 *μ*g C3G equivalent HB anthocyanin in 1 mL water) poured into the medication cup. The nebulizer was run at ≥0.35 mL/min flow rate at room temperature for 21 min to aerosolize each treatment. The total anthocyanin dose for each mouse per day was 28 *μ*g C3G equivalent anthocyanin. Mice were carefully observed during the inhalation treatment for distress signs. They were exposed to the inhalation treatment for six days with two days intervals between each treatment (Figure 1(c)). At the end of the experiment, mice were anesthetized and sacrificed by cervical dislocation. Lungs were excised and stored at -80°C.

#### 2.8.2. UPLC/ESI/Q-TOF-MS Analysis

The whole lungs from each mouse were weighed and homogenized in PBS (1 : 5, w/v) in an ice bath. The homogenate was centrifuged at 14,000×*g* for 15 min at 4°C, and the supernatant was collected. An aliquot (500 *μ*L) of the homogenate was mixed with two parts of cold-acetonitrile and acetone (80 : 20, v/v) containing the internal standard and stored at 4°C overnight to enhance protein precipitation. The mixture was centrifuged at 14,000×*g* for 15 min at 4°C; the supernatant was collected and concentrated under a gentle stream of nitrogen gas. The resultant was reconstituted in methanol, and the ultrahigh-performance liquid chromatography-electrospray ionization quadrupole time-of-flight mass spectrometry (UPLC/ESI/Q-TOF-MS) was used for metabolite analysis.

### 2.9. Statistical Analysis

All the experiments were performed in three independent experiments and analyzed by one-way analysis of variance (ANOVA) using Tukey's post hoc test and Bonferroni test using GraphPad Prism 5 software (GraphPad Software Inc., San Diego, CA, USA). The *in vivo* experiment had three treatment groups with five mice in each group. Data were presented as mean ± standard deviation (SD), and *p* ≤ 0.05 was considered significant between experimental groups.

## 3. Results

### 3.1. Physical and Chemical Characteristics of Nanoparticles

**Figure 2:**
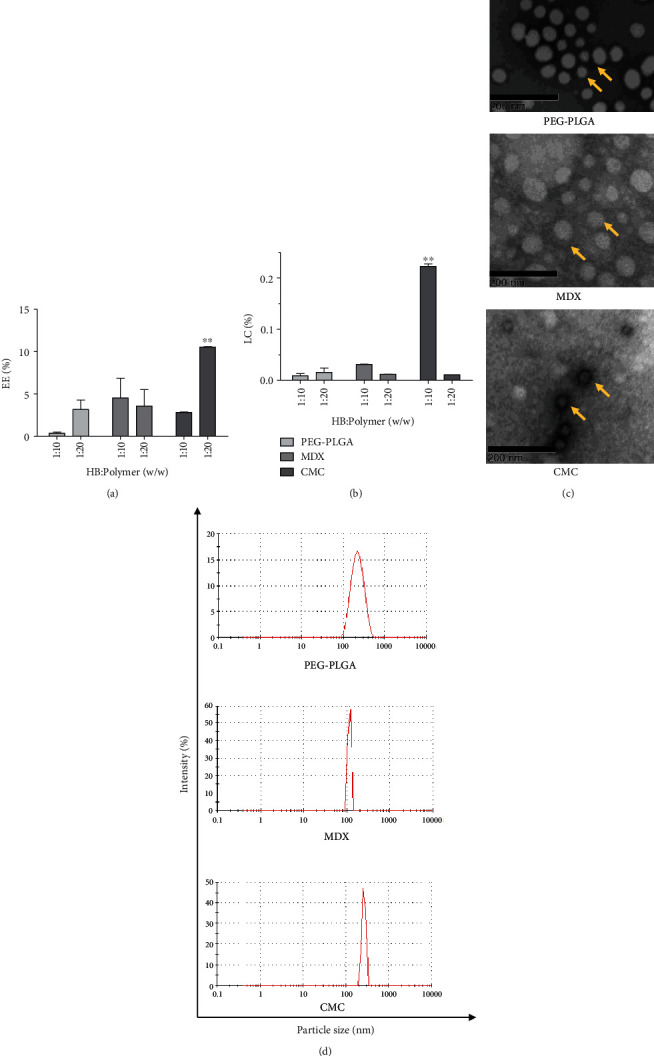
The physical and chemical characteristics of HB anthocyanin-encapsulated nanoparticles. (a) The percentage anthocyanin encapsulation efficiency (EE), and (b) the percentage loading capacity (LC) at HB: polymer (w/w) ratio of 1 : 10 and 1 : 20. (c) Transmission electron microscopy images of the anthocyanin-loaded nanoparticles at 1 : 10 HB: polymer ratio. Yellow color arrows indicate the spherical shape of nanoparticles. (d) The particle size distribution of HB anthocyanin-encapsulated nanoparticles at 1 : 10 HB: polymer ratio. The results represent three independent experiments. One-way analysis of variance was performed (*p* < 0.0001) with Tukey's pairwise comparison (at *α* = 0.01) for mean comparison. ^∗∗^ indicates statistical difference at *p* ≤ 0.01 with mean ± SD. Scale bar =200 nm. PEG-PLGA : polyethylene glycol-poly(lactide-co-glycolide); MDX: maltodextrin; CMC: carboxymethyl chitosan.

The pH differential method was used to estimate the anthocyanin encapsulation efficiency and the loading capacity in PEG-PLGA, MDX, or CMC polymer-based nanoparticle at 1 : 10 and 1 : 20 anthocyanin: polymer ratios. There was a statistically significant interaction between the type of polymer and polymer: HB ratio on anthocyanin encapsulation efficiency (*F* = 5.428, *p* = 0.0048) and loading capacity (*F* = 172.4, *p* < 0.0001). The encapsulation efficiency was significantly different (*p* = 0.033) among each polymeric nanoparticle and significantly high in CMC at 1 : 20 ratio (10% ± 0.1, *p* < 0.01, Figure 2(a)). Similarly, the anthocyanin loading capacity was significantly high in CMC (*p* < 0.001) at the 1 : 10 anthocyanin: polymer ratio (Figure 2(b)). The anthocyanin: polymer mixing ratio had a greater influence on encapsulation efficiency (*p* = 0.0012) and loading capacity (*p* < 0.001). In all three polymers, anthocyanin loading capacity was less than 1%. The HB anthocyanin-encapsulated PEG-PLGA, MDX, and CMC particles (1 : 10) were observed under a transmission electron microscope (TEM) to visualize their shape. All the tested nanoparticles had smooth spherical-shaped morphology (Figure 2(c)). The nanoparticles were <450 nm in size; however, anthocyanin-loaded CMC nanoparticles were <200 nm (Figure 2(d)). The particle distribution intensity was less than one for all the nanoparticles; however, the CMC nanoparticles had a high particle distribution intensity (0.7–0.9). The zeta potential of the prepared nanoparticles varied depending on the polymer characteristics. For example, CMC nanoparticles had a positive zeta potential, while PEG-PLGA and MDX nanoparticles showed a negative zeta potential. The TPC and the antioxidant capacity were significantly higher in anthocyanin-encapsulated CMC nanoparticles than in the other polymers (Table 1). According to these findings, CMC was selected as the most suitable candidate for HB-anthocyanin encapsulation and tested *in vitro* and *in vivo*.

### 3.2. Characteristics of the CMC Nanoparticles

**Figure 3:**
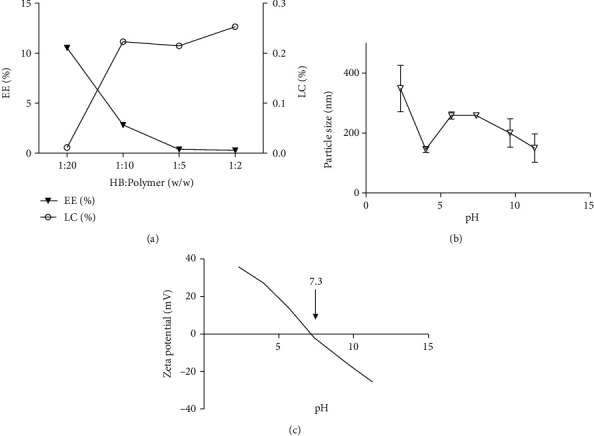
The physical and chemical characteristics of HB-CMC nanoparticles. (a) The effect of HB: CMC (w/w) ratio on the percentage anthocyanin encapsulation efficiency (EE) and the percentage loading capacity (LC). (b) The effect of pH on particle size, and (c) zeta potential of HB-CMC nanocarriers. The CMC nanoparticles are electrically neutral at pH 7.3. The results represent three independent experiments. HB: haskap berry anthocyanin; CMC: carboxymethyl chitosan.

In CMC nanoparticles, the loading capacity was significantly higher (*p* < 0.001) at 1 : 5 and 1 : 10 ratios than at the 1 : 20 anthocyanin: polymer ratio. The anthocyanin: CMC ratio had a negative effect on encapsulation efficiency, while it showed a positive effect on the loading capacity (Figure 3(a)). The particle size and the zeta potential were reduced at alkaline pH (Figures 3(b) and 3(c)). The isoelectric point of anthocyanin-encapsulated CMC nanoparticles was pH 7.3. In this study, all zeta potential values obtained at acidic pH were less than 12 mV, indicating the formation of aggregates in the dispersion conditions. Based on the anthocyanin encapsulation efficiency, loading capacity, and morphological features, CMC at 1 : 10 ratio was chosen as the best anthocyanin: polymer ratio to encapsulate the anthocyanins and used *in vitro* and *in vivo* experiments.

### 3.3. The Cytoprotective Effect of HB-CMC Nanoparticles in BEAS-2B Cells

The effect of the E-CMC and HB-CMC nanoparticles was initially studied in human BEAS-2B cells using the MTS assay. The effect of nanoparticles on BEAS-2B cells treated with NNKOAc, a carcinogen that induced oxidative stress and DNA damage in BEAS-2B cells at 100 *μ*M dose (3 h), was also determined.

#### 3.3.1. Cell Morphology and Viability

**Figure 4:**
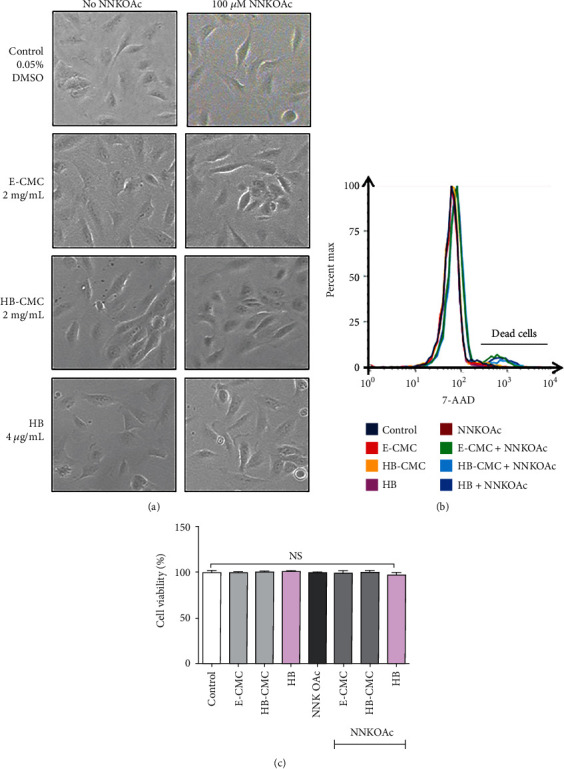
The cytoprotective effect of empty carboxymethyl chitosan (CMC) nanoparticles (E-CMC), HB encapsulated with CMC (HB-CMC), and an equivalent dose of free HB-anthocyanin (HB) against carcinogenic NNKOAc in BEAS-2B cells. Cytotoxicity was determined by observing alterations in phenotype (3(a)) and cell viability (3(b) and 3(c)). (a) BEAS-2B cells were treated with 2 mg/mL E-CMC, HB-CMC (comprised 4 *μ*g/mL C3G equivalent anthocyanin), or 4 *μ*g/mL HB-anthocyanin for 3 h separately, followed by exposure to 100 *μ*M NNKOAc for another 3 h. (a) The morphology of BEAS-2B cells was not different among vehicle control and treatment groups. Images were taken at 100× magnification using the inverted phase-contrast microscope. (b) The cell viability was determined by 7-AAD flowcytometric assay on 10,000 events. The cell population to the left of the graph represents live cells. (c) All the treatments maintained over 90% cell viability and did not significantly differ from the control. The results represent three independent experiments. One-way analysis of variance was performed (*p* < 0.0001) with Tukey's pairwise comparison (at *α* = 0.05) for mean comparison. NS: results do not significantly different; E-CMC: anthocyanin-free carboxymethyl chitosan nanoparticles; HB-CMC: haskap berry anthocyanin encapsulated nanoparticles; HB: haskap berry anthocyanin; DCFDA: 2,7-dichlorofluorescein diacetate; C3G: cyanidin-3-*O*-glucoside.

BEAS-2B cells were treated with nanoparticles (25–400 *μ*g/mL) or HB anthocyanin for 24 h, after which cell morphology was assessed, and cell viability was measured. The morphology of BEAS-2B cells showed epithelial characteristics and was not different among vehicle control and all the treatment groups (Figure 4(a)). Signs of cell apoptosis, such as membrane blebbing and shrinkage of cells, were not observed in any treatment. All the treatments had over 90% cell viability and did not show significant (*p* > 0.05) cell death (Figures 4(b) and 4(c)).

#### 3.3.2. Carcinogen-Induced Oxidative Stress

**Figure 5:**
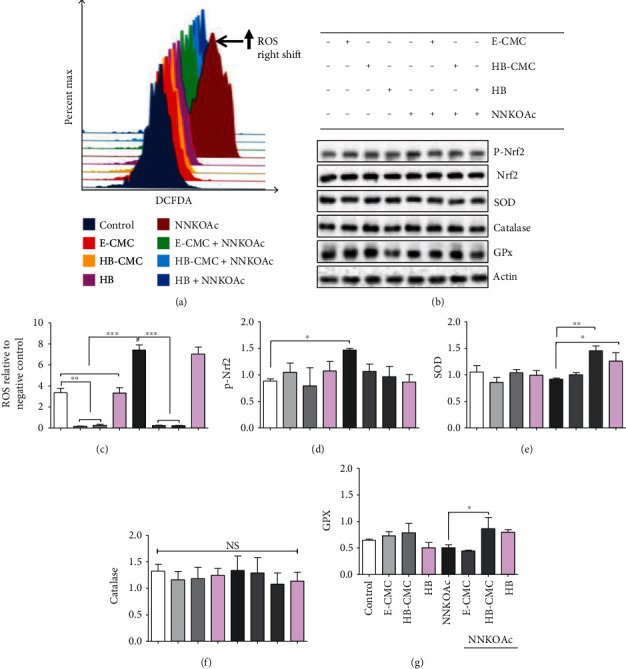
The effect of carboxymethyl chitosan (CMC) nanoparticles (E-CMC), HB encapsulated with CMC (HB-CMC), and an equivalent dose of free HB-anthocyanin (HB) against NNKOAc-induced oxidative stress in BEAS-2B cells. BEAS-2B cells were treated with 2 mg/mL E-CMC, HB-CMC (comprised 4 *μ*g/mL C3G equivalent anthocyanin), and 4 *μ*g/mL HB-anthocyanin for 3 h separately, following exposure to 100 *μ*M NNKOAc for another 3 h. (a) Intracellular reactive oxygen species (ROS) levels were assessed by measuring the dichlorofluorescein status in treated cells by flow cytometry. The CMC nanoparticles reduced (graphs skewed towards left) the NNKOAc-induced ROS generation (skewed towards the right). (b) The expression of p-Nrf2 and antioxidant enzymes, determined by western blot. (c–g) The bar graphs present the quantitative data of the DCFDA flow cytometry data and the expression of p-Nrf2, superoxide dismutase (SOD), catalase, and glutathione peroxidase (GPx). The results represent three independent experiments. One-way analysis of variance was performed (*p* < 0.0001) with Bonferroni pairwise comparison (at *α* = 0.1) for mean comparison. ^∗^, ^∗∗^, and ^∗∗∗^ indicate statistical differences at *p* ≤ 0.1, 0.01, and 0.001, respectively, with mean ± SD. NS: results do not significantly different; E-CMC: anthocyanin-free carboxymethyl chitosan nanoparticles; HB-CMC: haskap berry anthocyanin encapsulated nanoparticles; HB: haskap berry anthocyanin; DCFDA: 2,7-dichlorofluorescein diacetate; C3G: cyanidin-3-*O*-glucoside.

The E-CMC and HB-CMC nanoparticles reduced cellular reactive oxygen species (ROS) and the NNKOAc-induced ROS generation, but the same dose of HB anthocyanin (4 *μ*g/mL) did not decrease the NNKOAc-induced ROS in BEAS-2B cells (Figures 5(a) and 5(c)). Neither CMC nanoparticles nor HB anthocyanin (4 *μ*g/mL) significantly (*p* > 0.05) altered the expression of phosphorylated Nrf2 and antioxidant enzymes, SOD, catalase, and GPx, (Figures 5(b) and 5(d)–5(g)) in BEAS-2B cells. NNKOAc induced the phosphorylation of Nrf2(S40). NNKOAc did not change the expression of catalase enzyme in BEAS-2B cells but suppressed SOD and GPx expression by 13% and 20%, respectively, compared to the control. The E-CMC and HB-CMC pretreatment suppressed the NNKOAc-induced Nrf2 phosphorylation by 30% in BEAS-2B cells. The HB-CMC pretreatment restored the expression of SOD and GPx in NNKOAc-exposed BEAS-2B cells. HB-CMC nanoparticles and HB anthocyanin pretreatment reduced the expression of catalase by 10% in comparison to the NNKOAc treatment alone. Therefore, the observed cytoprotective effect of nanoparticles against ROS-mediated cytotoxicity could be partially by activating antioxidant enzymes, such as SOD and GPx, direct scavenging of ROS, or through another mechanism.

#### 3.3.3. Carcinogen-Induced DNA Damage

**Figure 6:**
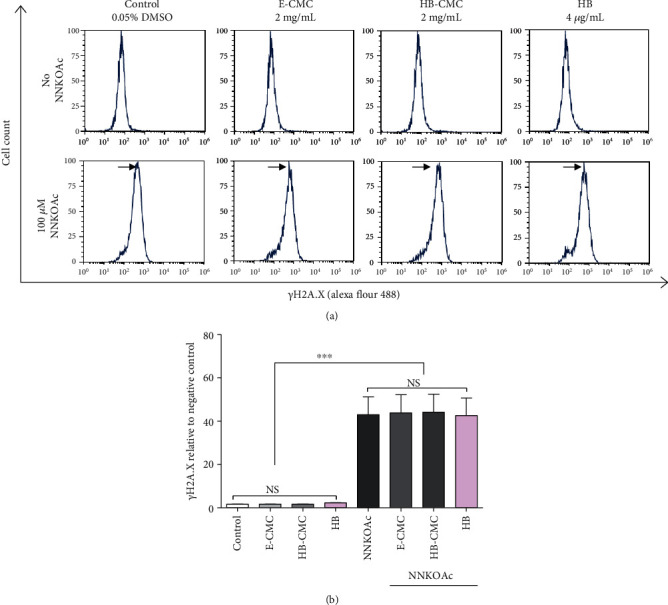
The protective effect of carboxymethyl chitosan (CMC) nanoparticles (E-CMC and HB-CMC) and an equivalent dose of free HB-anthocyanin (HB) against NNKOAc-induced DNA double-strand break in BEAS-2B cells. The protective effect was measured by *γ*H2A.X assay. BEAS-2B cells were treated with 2 mg/mL E-CMC, HB-CMC (comprised 4 *μ*g/mL C3G equivalent anthocyanin), and 4 *μ*g/mL HB-anthocyanin for 3 h separately, following exposure to 100 *μ*M NNKOAc for another 3 h. (a) The treated cells were fixed, stained with primary antibody for *γ*H2A.X foci and then incubated with secondary antibody anti-rabbit Alexa Fluor 488 before being measured by flow cytometry on 10,000 events. Top panel: neither nanoparticle nor HB induced DNA damage in BEAS-2B cells at studied doses. Lower panel: the treatments did not reduce the NNKOAc-induced double-strand breaks (skewed graph right, indicated by black arrowheads) in BEAS-2B cells. (b) The nanoparticles or HB did not reduce the NNKOAc-induced DNA double-strand breaks in BEAS-2B cells. One-way analysis of variance was performed (*p* < 0.0001) with Tukey's pairwise comparison (at *α* = 0.05) for mean comparison. ^∗∗∗^ indicate statistical difference at *p* ≤ 0.001. NS: results do not significantly different.

**Figure 7:**
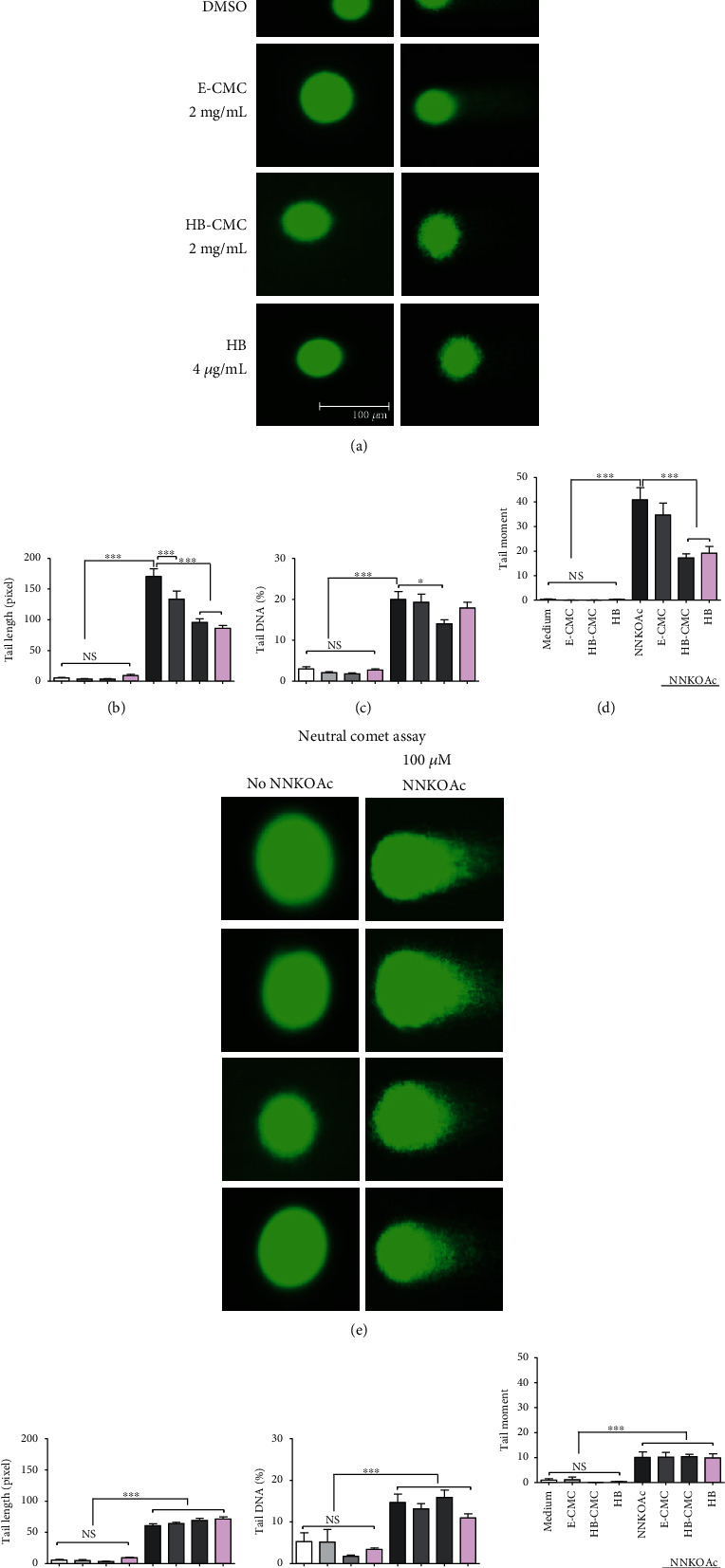
The protective effect of carboxymethyl chitosan (CMC) nanoparticles (E-CMC and HB-CMC) and an equivalent dose of free HB-anthocyanin (HB) against NNKOAc-induced DNA damage in BEAS-2B cells. The protective effect was measured by alkaline and neutral comet assays. BEAS-2B cells were treated with 2 mg/mL E-CMC, HB-CMC (comprised 4 *μ*g/mL C3G equivalent anthocyanin), and 4 *μ*g/mL HB-anthocyanin for 3 h separately, following exposure to 100 *μ*M NNKOAc for another 3 h. (a) Alkaline comet mages represent both single- and double-strand breaks. The HB-CMC nanoparticles significantly reduced NNKOAc-induced tail length (b), tail DNA percentage (c), and tail moment (d) as quantified by alkaline comet assay. (e) Neutral comet images represent the DNA double-strand breaks. The HB-CMC nanoparticles did not attenuate the NNKOAc-induced tail length (B), tail DNA percentage (c), and tail moment (d) as quantified by neutral comet assay. Nuclear DNA comets were stained with SYBR Green I dye, and images were taken by fluorescence microscope. The scale =100 *μ*m. The comet tail length, percent DNA in tail, and tail moment (tail moment = measure of tail length × measure of DNA in the tail) were quantified in (30–100 nuclei from each treatment) using Fiji ImageJ and Open Comet Version 1.3 software. The results represent three independent experiments (*n* = 3). One-way analysis of variance was performed (*p* < 0.0001) with Tukey's pairwise comparison (at *α* = 0.05) for mean comparison. ^∗^ and ^∗∗∗^ indicate statistical differences at *p* ≤ 0.1 and 0.001, respectively, with mean ± SD. NS: results do not significantly different.

None of the nanoparticles or HB anthocyanin (4 *μ*g/mL) induced DNA damage in BEAS-2B cells, whereas NNKOAc increased DNA double-strand breaks in the BEAS-2B cells. The nanoparticles or HB anthocyanin did not reduce the NNKOAc-induced DNA double-strand breaks in BEAS-2B cells. The *γ*H2A.X (Figures 6(a) and 6(b)) and neutral comet assays (Figures 7(e)–7(h)) data had a similar pattern regarding the double-strand breaks. In contrast, HB-CMC nanoparticles and HB anthocyanin significantly reduced (*p* < 0.05) NNKOAc-induced DNA damage, as measured by alkaline comet assay (Figures 7(a)–7(d)). Tail length and tail DNA percentage were significantly reduced (*p* < 0.05) in cells pretreated with HB-CMC nanoparticles compared to NNKOAc-exposed cells. The alkaline comet data represent both single- and double-strand breaks. BEAS-2B cells in neutral comet assay showed shorter tails than the alkaline comet images (Figures 7(a) vs 7(e)).

### 3.4. The Bioavailability of Anthocyanins in the Lungs

**Figure 8:**
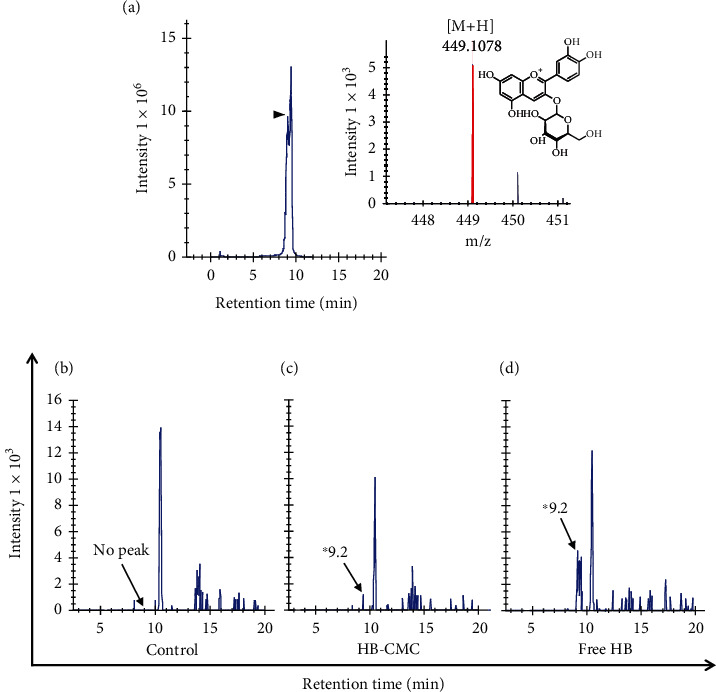
Detection of cyanidin-3-*O*-glucoside (C3G), the major anthocyanin present in HB in lung tissue of A/JCr mice which were given pulmonary delivery of nanoparticles or HB extract. Nanoparticles (E-CMC and HB-CMC) and HB extracts were dissolved separately in sterilized deionized water (4 *μ*g C3G equivalent HB anthocyanin in 1 mL water) aerosolized by an ultrasonic mesh nebulizer to facilitate passive inhalation through a nose-only exposure device. The inhalation treatment (28 *μ*g C3G equivalent nanoparticles/mouse/day) was given for six days, leaving two days gap between each treatment, and lung tissues were collected and analyzed by UPLC/ESI/MS-O-TOF for anthocyanin metabolites. C3G was detected after 9.2 min in HB-CMC and HB extract-treated mice lung tissue samples. Chromatograms shown are composites C3G standard (a), lung tissue from mice of control (E-CMC) (b), HB-CMC (c), and HB extract inhalation (d). E-CMC: anthocyanin-free carboxymethyl chitosan nanoparticles; HB-CMC: HB anthocyanin-encapsulated nanoparticles; HB: haskap berry anthocyanin.

C3G, the major anthocyanin in HB, and its metabolites were observed in the lung tissues after inhalation of HB-CMC nanoparticles or an equivalent dose of C3G from HB. Interestingly, after one hour of inhalation treatment, C3G was found in the lungs of mice that inhaled HB-CMC nanoparticles or free HB anthocyanin (Figure 8). The primary C3G metabolites detected were cyanidin (only in HB-CMC nanoparticle-treated mice), cyanidin-3-*O*-glucuronide, and peonidin-3-*O*-glucoside (only in free HB-treated mice). The protocatechuic acid was found in all the treatment groups, including the control group (Table 2). Furthermore, anthocyanin-derived phenolic acids, such as hippuric acid and caffeic acid, were also observed in the lungs of mice exposed to HB.

## 4. Discussion

Anthocyanins are readily degraded and oxidized to various forms due to the oxidation of their hydroxyl groups. The HB comprises C3G, peonidin-3-*O*-glucoside, malvidin-3-*O*-glucoside, cyanidin-3-*O*-rutinoside, and many other phenolic acids, such as chlorogenic acid, ferulic acid, and caffeic acid. C3G is the most predominant, representing about 90% of the anthocyanins in HB [12]. The stability of anthocyanins can be enhanced by conjugating them with macromolecules, such as polysaccharides, polymers, or proteins [28]. We used synthetic (PEG-PLGA) and natural (MDX and CMC) polymers to coat HB anthocyanin to prolong and enhance lung bioavailability. The encapsulation efficiency of anthocyanins, either as nanoparticles or microparticles, ranges from 3 to 60% and depends on factors such as polymer characteristics, anthocyanin to polymer ratio, conditions at processing (pH, time, and solvents), and method of encapsulation [14, 29, 30]. CMC showed a higher HB anthocyanin encapsulation efficiency and loading capacity among the selected polymers. The lower encapsulation efficiency in MDX and PEG-PLGA (<5%) in the present study may be due to the long polymer chain of MDX and/or the hydrophobic nature of PEG-PLGA (5,000 : 7,000). It is reported that chain length affects the formation, stability, and entrapment of molecules in MDX nanoparticles. MDX with low dextrose equivalent values (4.0–7.0) has long chains that aggregate quickly, resulting in poor anthocyanin content compared to the short-chain MDX (dextrose equivalent value >13) [31, 32]. Additionally, PEG-PLGA (amphiphilic) in DCM creates a hydrophobic environment that negatively affects the solubility of hydrophilic HB anthocyanins, resulting in weak binding capacity with PEG-PLGA di-block polymer. The PEG: PLGA ratio also affects the drug loading efficiency [33]. In the present study, the longer MDX polymeric chains and hydrophobic PEG-PLGA solution may have reduced the anthocyanin encapsulation efficiency and loading capacity.

**Figure 9:**
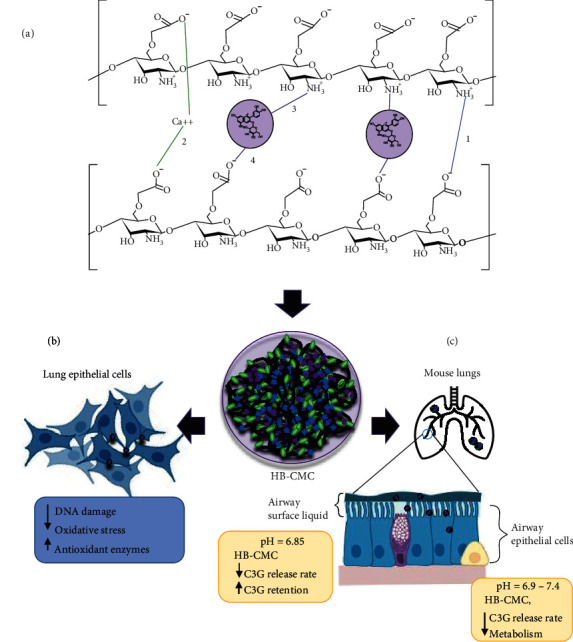
Schematic illustration of the HB-CMC nanoparticle formation and its cytoprotective effect in lung epithelial cells *in vitro* and bioavailability in mice lungs after nose-only inhalation. (a) CMC polymers make cross-links (1) between each other (NH_3_^+^—COO^−^), (2) with Ca^2+^ (COO^−^—Ca^2+^—COO^−^), and (3) with C3G (COO^−^—C3G and NH_3_^+^—C3G) during ionic gelation. (b) HB-CMC protects against carcinogen-induced DNA damage and oxidative stress and induces antioxidant enzymes SOD and GPx in BEAS-2B lung epithelial cells. (c) The slightly acidic pH in airway surface liquid and cytoplasm of lung cells slow the C3G release rate from HB-CMC in the lung tissues leading to prolonged retention of intact C3G than from the free HB. References are cited in the text. The Biorender free software was used to draw the illustrations.

The loading capacity and the amount of drug-loaded per unit weight of the nanoparticle are essential in formulations as it determines the number of particles required for a given dose [34]. The inverse association between encapsulation efficiency and loading capacity in CMC may be due to the variation of the binding capacity of anthocyanin on the CMC-Ca matrix. The carboxylate ion (COO^−^) groups of CMC interact with divalent calcium ions (Ca^2+^) and form cross-links between polymers. Further, CMC polymers form stable bonds between NH_3_^+^ and COO^−^ groups. In anthocyanin mixed CMC-Ca matrix, anthocyanins form electrostatic interactions with NH_3_^+^ and COO^−^ groups of CMC (Figure 9(a) 1–4 bonds) [21, 35, 36]. It is further shown that anthocyanins can incorporate into CMC through its C=O group of the benzopyran aromatic ring [37]. The ability to form ionic interactions between anthocyanin and CMC may be the reason to show a higher encapsulation efficiency of HB anthocyanins in CMC-Ca matrix compared to PEG-PLGA and MDX. The deprotonation of CMC affects these interactions as it determines the number of reactive groups available on the polymer to interact with each other and with HB anthocyanins. For instance, at the 20 : 1 CMC: anthocyanin ratio, HB anthocyanin has enough ionic groups (on CMC) to bind, resulting in increased encapsulation efficiency but lower loading capacity due to the limitations of anthocyanins in the mixture. At the 5 : 1 CMC: anthocyanin ratio, ionic groups are limited for anthocyanins to bind, resulting in less encapsulation efficiency but higher loading capacity. Therefore, finding methods to increase the electrostatic interactions and ionic cross-links among C3G-CMC-Ca^2+^ ion matrices is necessary. Modifying CMC polymer by altering the number of NH_2_ groups and COOH on CMC and incorporating tripolyphosphate as the cross-linking agent may enhance the anthocyanin encapsulation efficiency and loading capacity.

The stability of the CMC nanoparticles is pH-dependent [21]. pH determines the protonation or deprotonation levels of NH_2_ (NH_3_^+^ > COO^−^ in acidic pH) and COOH groups (NH_3_^+^ < COO^−^ in alkaline pH) that finally determines the interactions between NH_3_^+^ and COO^−^ of CMC polymers during the synthesis of nanoparticles [21, 38]. The zeta potential, the electric potential at the shear plane in a suspension, determines the stability of particles [20]. The zeta potential of CMC nanoparticles was negative at pH >7.3, which is beyond the isoelectric point, but was positive below the isoelectric point. This effect may result from COO^−^ groups at higher pH and NH_3_^+^groups at lower pH [21]. Near the isoelectric point, the charges of NH4+ and COO^−^ groups are equal. The low polydispersity index (<0.7) indicates the formation of almost monodispersed nanoparticles in all the polymers except for MDX, which showed a broad size distribution (Figure 2(b)).

NNKOAc is a nicotine-derived nitrosamine ketone (NNK) derivative in smoke and smokeless tobacco. NNK is a group I lung carcinogen that has been reported to induce oxidative stress and gene mutation both *in vivo* and *in vitro*. It has been reported that NNK generates superoxide anions and hydrogen peroxide [39]. Our previous studies have confirmed NNKOAc-induced oxidative stress in normal lung epithelial cells. NNKOAc also initiates DNA double-strand breaks in lung epithelial cells [12, 40]. Therefore, NNKOAc treatment was chosen to test the cytoprotective ability of HB-CMC nanoparticles on lung epithelial cells *in vitro*.

The present study shows that CMC nanoparticles with and without HB anthocyanins were not cytotoxic to normal lung epithelial cells *in vitro*. Furthermore, 2 mg/mL of CMC nanoparticles reduced the carcinogen-induced oxidative stress but not the DNA double-strand breaks. It is reported that CMC, conjugated to drugs and unconjugated in free form, can regulate the Nrf2/ARE pathway and attenuate the activity of antioxidant enzymes SOD, catalase, GPx, and DNA damage against oxidative stress *in vitro* and *in vivo* [40–44]. Similarly, we found that E-CMC and HB-CMC can reduce ROS accumulation and restore the expressions of SOD and GPx in carcinogen-exposed BEAS-2B cells. However, no significant effect was found on the expression of the catalase enzyme. The HB-CMC and free HB anthocyanin improved the expression of SOD and GPx, in carcinogen-exposed cells. The FRAP and DPPH• radical scavenging antioxidant capacity assays demonstrated HB-CMC particles in scavenging free radicals. The ROS scavenging ability of CMC is reported. The hydroxyl and amino groups of CMC scavenge superoxide and hydroxyl radicals and reduce oxidative stress [45–47]. Therefore, ROS mediating characteristics of CMC nanoparticles may be due to the activation of SOD and GPx to a certain extent, direct scavenging of NNKOAc-induced oxidative radicals, and activating nonenzymatic antioxidants such as glutathione.

DNA double-strand breaks in BEAS-2B cells were detected by *γ*H2A.X immunofluorescence and neutral comet assays. At the selected doses, E-CMC, HB-CMC, and HB anthocyanins did not induce DNA damage. However, none of the nanocarriers protected the cells from NNKOAc-induced DNA double-strand breaks. Increasing the HB-CMC treatment period (>3 h) and treatment dose in future studies alter the effect of HB-CMC nanoparticles' protective effect on NNKOAc-induced genotoxicity in BEAS-2B lung epithelial cells. The neutral comet assay detects double-strand breaks, while the alkaline comet assay measures both single- and double-strand breaks, alkali-labile sites, and DNA-DNA cross-linking. Alkaline comet assay (pH 13) is more sensitive to a minor degree of DNA damage [27]. The results of an alkaline comet assay suggest that NNKOAc also can induce single-strand breaks and/or alkali-labile sites in BEAS-2B cells. The single-strand breaks and alkali-labile sites are formed by ROS, particularly hydroxyl radicals and singlet oxygen species [48]. NNK produces hydroxyl radicals and superoxide radicals that later convert into hydroxyl radicals. The protective effect in the alkaline comet assay may be due to the reduction of oxidative stress-induced single-strand breaks and alkali-labile sites in BEAS-2B cells (Figure 9(b)).

The presence of anthocyanins in mouse lung tissues following high dose (100 and 500 mg/kg body weight) oral administration has been reported. However, after 15 min of ingestion, most anthocyanins are lost from plasma and excreted through urine, resulting in low bioavailability [49]. In the present study, we delivered HB-anthocyanins (encapsulated form and free HB) at a lower dose (28 *μ*g C3G equivalent anthocyanin/mouse/days) directly into the mice lungs by facilitating passive inhalation. C3G, the major anthocyanin of HB, was detected in mouse lung tissues even after one hour of inhalation treatment. The results suggest that direct delivery to lung tissues prolongs the anthocyanin tissue retention and enhances tissue-specific bioavailability. Furthermore, cyanidin, the aglycone of C3G (only in HB-CMC treatment), peonidin-3-*O*-glucoside, and cyanidin-3-*O*-glucuronide (only in free HB treatment) were observed in lung tissues suggesting that lung cells can metabolize C3G into cyanidin and facilitate its phase 2 metabolism and/or part of HB-CMC and HB anthocyanins has reached the systemic circulation and undergone phase 2 liver metabolism within one hour of inhalation treatment. The absence of C3G-derived anthocyanins, such as peonidin-3-*O*-glucoside and cyanidin-3-*O*-glucuronide, in the lungs of HB-CMC nanoparticle-treated mice, indicates the protective effect of CMC envelope against enzymatic digestion of entrapped anthocyanins in the lungs. It is known that CMC has higher mucoadhesive characteristics, which facilitate active molecule absorption [48].

Additionally, the anthocyanin release rate from CMC nanoparticles is pH-dependent. For instance, the C3G release rate from C3G encapsulated CMC-Ca particles is higher (60% over six days period) under acidic conditions (pH 5.3) compared to the slightly acidic or neutral pH (20–30% over six days, pH 6-8–7.4) solutions [35]. CMC has low solubility at higher pH [50]. The mouse lung pH values range from 6.9 to 7.2 [51]. The slightly acidic environment in the mouse lung may have increased the stability of HB-CMC nanoparticles, resulting in a slower C3G release rate in mouse lungs. Hence, we only found C3G and aglycone cyanidin in HB-CMC-treated lungs. In humans, airway surface liquid/mucosa is slightly acidic (pH 6.85), and epithelial cell cytoplasm is slightly acidic or neutral (6.9–7.4) [51, 52]. These conditions may facilitate extended retention of HB-CMC nanoparticles and sustained C3G release into the lung tissues (Figure 9(c)). Lung diseases can alter the pH of the airway mucosa. For example, chronic bronchitis and rhinitis create slightly alkaline (pH 7–8) conditions, while some bacterial infections and lung cancer result in an acidic environment in the lungs (pH 5–6.5) [51]. These conditions favor the retention stability and prolong the release of C3G from HB-CMC in lung tissues. HB-CMC could therefore be a potential therapeutic to treat lung diseases, particularly cancer. However, it is crucial to quantify the time cause of the release of anthocyanins and their possible metabolites in lung tissues and plasma to determine the systemic distribution of anthocyanins after pulmonary delivery.

## 5. Conclusions

To improve the bioavailability and direct delivery of C3G-rich HB anthocyanins, three polymers, PEG-PLGA, MDX, and CMC, were investigated as the coating material. HB anthocyanin was successfully encapsulated by ionic gelation in CMC with Ca^2+^ ion as the cross-linker, forming spherical nanoparticles. The anthocyanin: polymer ratio affects the encapsulation efficiency and loading capacity of anthocyanins in CMC. *In vitro* studies in normal lung epithelial cells showed that nanoparticles can attenuate carcinogen-induced oxidative stress by restoring antioxidant enzymes and protecting against DNA damage. Pulmonary delivery of an extremely low dose of encapsulated anthocyanins extended the retention of intact C3G and delayed its tissue metabolism. Therefore, we suggest that direct delivery of anthocyanins to lung tissues has therapeutic potential to treat various lung diseases, including cancer.

## Figures and Tables

**Table 1:** The physical and chemical properties of anthocyanin-loaded nanoparticles prepared using PEG-PLGA, MDX, or CMC.

Encapsulant	Core content	Particle size (nm)	PDI	Zeta potential (mV)	Total yield (%)	TPC (mg GAE/g DW)	FRAP (*μ*M TE/g DW)	IC_50_ (mg/mL DW)
CMC	Empty	97–322	0.9 ± 0.1	35.0 ± 1.8	65 ± 4	—	—	—
	HB	163–170	0.7 ± 0.0	30.4 ± 6.6	63 ± 5	6.4 ± 0.3	1766 ± 23	5.3 ± 1

MDX	Empty	50–465	0.6 ± 0.1	−0.5 ± 0.1	100 ± 0	—	—	—
	HB	116-420	0.6 ± 0.1	−0.5 ± 0.2	94 ± 1	2.1 ± 0.0	793 ± 24	ND

PEG-PLGA	Empty	120-310	0.5 ± 0.1	−1.1 ± 0.1	19 ± 3	—	—	—
	HB	110-280	0.4 ± 0.0	−4.2 ± 0.0	35 ± 2	0.02	229 ± 14	ND

Data represents nanoparticles HB: polymer ratio of 1 : 10. Total yield (%) = weight of nanoparticles/weight of the material used to prepare nanoparticles ×100. PEG-PLGA : polyethylene glycol-poly(lactide-co-glycolide); MDX: maltodextrin; CMC: carboxymethyl chitosan; PDI: particle distribution intensity; TPC: total phenolic content; FRAP: ferric reducing antioxidant power assay; DW: dry weight; empty: anthocyanin-free nanoparticles; HB: haskap berry extract; GAE: gallic acid equivalent; TE: Trolox equivalent; ND: not detected.

**Table 2:** The identified anthocyanins and their metabolites in lung tissues of A/JCr mice following inhalation treatment.

Compound	m/z	Rt (min)	Mass error (ppm)	Inhalation treatment		
				Control (E-CMC)	HB-CMC	Free HB
	[M + H]^+^					
Cyanidin-3-*O*-glucoside	449.1078	9.2	0	—	^∗^	^∗^
Cyanidin	287.0550	16.44	0.6	—	^∗^	—
Cyanidin-3-*O*-glucuronide	463.0871	16.16	0.5	—	—	^∗^
Peonidin-3-*O*-glucoside	463.1235	15.5	0.6	—	—	^∗^
	[M-H]^−^					
Protocatechuic acid	153.019	5.9		^∗^	^∗^	^∗^
Hippuric acid	178.051	8.6		—	^∗^	^∗^

m/z: mass/charge; Rt: retention time in minutes; control: mice given anthocyanin-free carboxymethyl chitosan (E-CMC) nanocarriers; HB-CMC: haskap berry anthocyanin encapsulated carboxymethyl chitosan nanoparticles; HB: haskap berry extract; ^∗^indicates metabolites detected using high-resolution mass spectrometry (UPLC/ESI/MS-Q-TOF); and – metabolites not detected.

## Data Availability

All the data are presented in the manuscript.

## Abbreviations

C3G: Cyanidin-3-*O*-glucoside
HB: Haskap berry
CMC: Carboxymethyl chitosan
MDX: Maltodextrin
PEG-PLGA: Polyethylene glycol-poly(lactide-co-glycolide)
ROS: Reactive oxygen species
EDTA: Ethylenediaminetetraacetic acid
E-CMC: Anthocyanin-free carboxymethyl chitosan
HB-CMC: Haskap berry anthocyanin encapsulated carboxymethyl chitosan
NNK: 4-(Methylnitrosamino)-1-(3-pyridyl)-1-butanone
NNKOAc: 4-[(Acetoxymethyl) nitrosamino]-1-(3-pyridyl)-1-butanone
UPLC/ESI/MS-Q-TOF: Ultra-performance liquid chromatography quadrupole time-of-flight mass spectrometry
SOD: Superoxide dismutase
GPX: Glutathione peroxidase
*γ*H2A.X: Phosphorylated histone H2A.X at serine 139
Nrf2: Nuclear factor erythroid 2-related factor 2
PDI: Particle distribution intensity
TPC: Total phenolic content
FRAP: Ferric reducing antioxidant power assay
DW: Dry weight
GAE: Gallic acid equivalent
TE: Trolox equivalent
DPPH: 2,2-Diphenyl-1-picrylhydrazyl assay
TPTZ: 2,3,4-Tris(2-pyridyl)-s-triazine
Q3G: Quercetin 3-*O*-glucoside
DCFDA: 2',7'-Dichlorofluorescein diacetate
ND: Not detected.
